# From monetite plate to hydroxyapatite nanofibers by monoethanolamine assisted hydrothermal approach

**DOI:** 10.1038/s41598-018-33936-4

**Published:** 2018-10-18

**Authors:** Katarzyna Suchanek, Amanda Bartkowiak, Marcin Perzanowski, Marta Marszałek

**Affiliations:** The Institute of Nuclear Physics Polish Academy of Sciences, Radzikowskiego 152, 31-342 Krakow, Poland

## Abstract

Calcium phosphates offer outstanding biological adaptability. Thanks to their specific physico-chemical properties they are one of the most widely used materials in bone tissue engineering applications. The search for an innovative and economic strategy of synthesizing their different forms has been drawing considerable attention in the field. Herein, we report on a facile hydrothermal process in the presence of ethylenediamine tetraacetic acid and monoethanolamine to obtain various forms of calcium phosphates. The monoethanolamine served as an alkaline source and crystal growth modifier, while ethylenediamine tetraacetic acid was used to control the Ca^2+^ supersaturation level under high temperature and high pressure conditions. The obtained inorganic compounds were examined for their elemental composition, morphology, and structure using scanning electron microscopy, Raman spectroscopy, and powder x-ray diffraction. We were able to selectively synthesize monetite plate-like microcrystals as well as hydroxyapatite plates and nanofibers by simply varying the concentration of monoethanolamine.

## Introduction

In the recent years, bone substitutes have been often used in dental and orthopaedic surgery^1^. Among the available materials, autografts are considered the gold standard for achieving strong chemical bonding with the living tissue due to their high biocompatibility after implantation^2^. However, the restraints in the autografts use, associated with their limited availability or donor site morbidity, especially chronic pain, impose a high demand for their alternatives in bone grafting.

Calcium phosphate-based biomaterials are one of the most promising materials for applications in bone replacement or regeneration because of their similarity to the mineral component of the bone^3–7^. Among calcium phosphates, hydroxyapatite (HAp, Ca_10_(PO_4_)_6_(OH)_2_) plays an important role thanks to its outstanding properties such as bioactivity, biocompatibility, and osteoconductivity. Due to their high biological activity and specific adsorbability towards various ions and organic molecules, hydroxyapatite-based materials have found application not only as artificial bones or scaffolds for tissue engineering but also as drug delivery carriers^8^, catalysts carriers^9^, and adsorbents^10,11^. Another calcium phosphate phase that has recently gained great attention is monetite, or dicalcium phosphate anhydrous (DCPA, CaHPO_4_). Besides its ability to regenerate the bone, monetite biomaterials can resorb *in vivo* faster than most of the calcium phosphates enabling the implant replacement by a newly formed tissue^12^. Monetite is an important component of some bone cements and is also used in toothpastes, chewing gums and the food processing industry^13^. Moreover, it has been used for a long time as a precursor phase in the synthesis of HAp^14^.

The efficiency of calcium phosphates in various applications strongly depends on their specific morphology, chemical composition, and particle size^15^. For instance, fibrous HAp is advantageous for adsorption and ion exchange due to the high specific surface area^16^, while for other applications where high mechanical strength is required rod-like crystals can be used as an reinforcement in the biocomposites^17^. Another example is nacre-like monetite which exhibits outstanding toughness, stiffness, and impact resistance^18^. Therefore, the development of new strategies for control of the morphology, chemical composition, and size of calcium phosphate crystals is of special significance and has become the object of intensive research.

The strategies to synthesize calcium phosphates are mainly divided into solid-state reactions and wet chemical methods. The latter include hydrolysis of salts^19,20^, sol–gel routes^21^, and hydrothermal processes^22–24^. Among them, hydrothermal method is an efficient and promising chemical route for obtaining various inorganic crystal architectures. The method has been applied in the synthesis of HAp crystals, in a form of nanoparticles, nanofibers, nanorods, plates^22,23^, as well as monetite crystals^24^. In the hydrothermal synthesis of various calcium phosphates, the precursor solution usually contains a calcium salt, a phosphate salt and a pH adjusting agent such as ammonia or urea^25^. In addition, other organic additives such as the coordination compounds (e.g. ethylenediamine tetraacetic acid (EDTA)) or the surfactants (e.g. cetyltrimethylammonium bromide (CTAB)) are intentionally introduced into the stock solution. These additives affect the final morphology of the produced particles^26^. Coordination agents regulate the degree of supersaturation of the solution by slow release of the calcium ions, whereas ionic surfactants influence the preferential adsorption of ions and molecules on the specific crystal planes. Such adsorption leads to the anisotropic crystal growth.

In the present work, we demonstrate a novel strategy for the synthesis of different forms of calcium phosphate crystals. The advance of our approach lies in the concomitant use of two reagents: EDTA and monoethanolamine (MEA), which serve as a complexing compound, and as a pH regulator and a modifier of the phase and shape of crystals, respectively. As far as we are aware, we are the first to use MEA to synthesize calcium phosphates through hydrothermal method. Cheng *et al*. have previously used MEA for hydrothermal synthesis of zinc oxide nanowires^27^. Here, we built on their results and expand them to calcium phosphates. MEA allows us to stabilize the initial solution across a wide pH range, and therefore expands the ability of the synthesis method to a broader selection of calcium phosphate phases. We find that by varying the concentration of MEA we are able to selectively synthesize monetite plate-like microcrystals as well as hydroxyapatite plates and nanofibers. Besides high crystallinity and structural purity of the produced materials, hydrothermal method offers simplicity and low-cost efficiency. We also hypothesize on the mechanism of the crystal growth under hydrothermal conditions in the presence of EDTA and, in particular, MEA.

## Results

### Structure and morphology of precipitates

**Figure 1:**
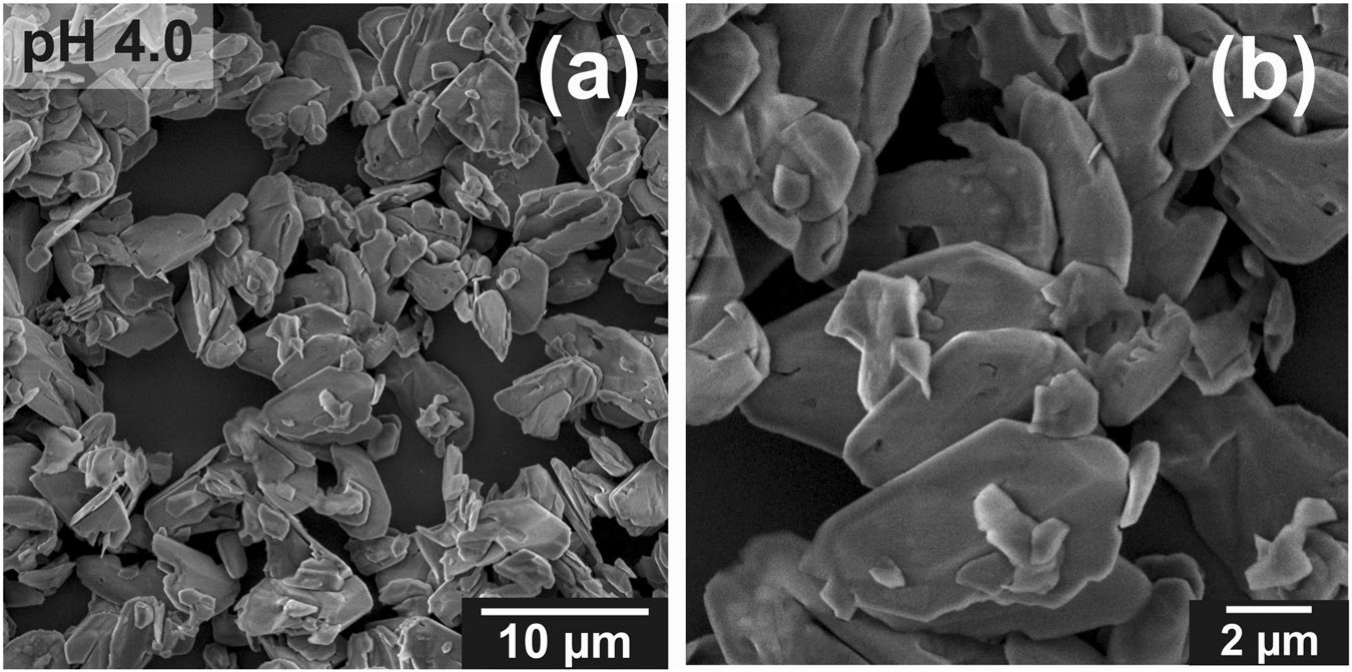
SEM images of precipitates obtained from solution with pH = 4.0 (SpH4.0). (**a**) Low and (**b**) high magnification SEM images.

**Figure 2:**
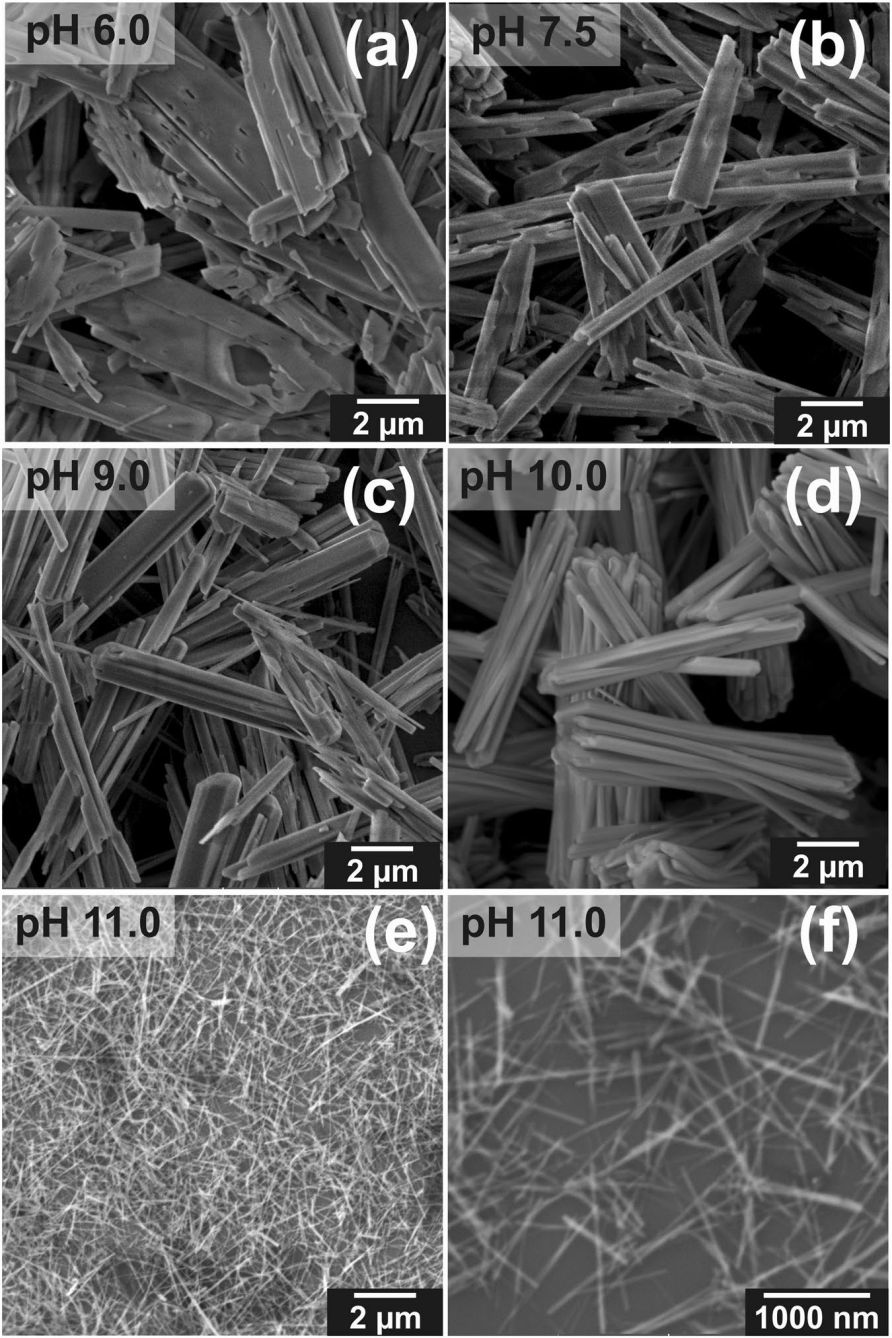
SEM images of particles with different final morphologies obtained through EDTA/MEA-assisted hydrothermal synthesis at different pHs: (**a**) pH = 6.0 (SpH6.0), (**b**) pH = 7.5 (SpH7.5), (**c**) pH = 9.0 (SpH9.0), (**d**) pH = 10.0 (SpH10.0). (**e**) Low and (**f**) high magnification SEM images of precipitates obtained from solution with pH = 11.0 (SpH11.0).

**Figure 3:**
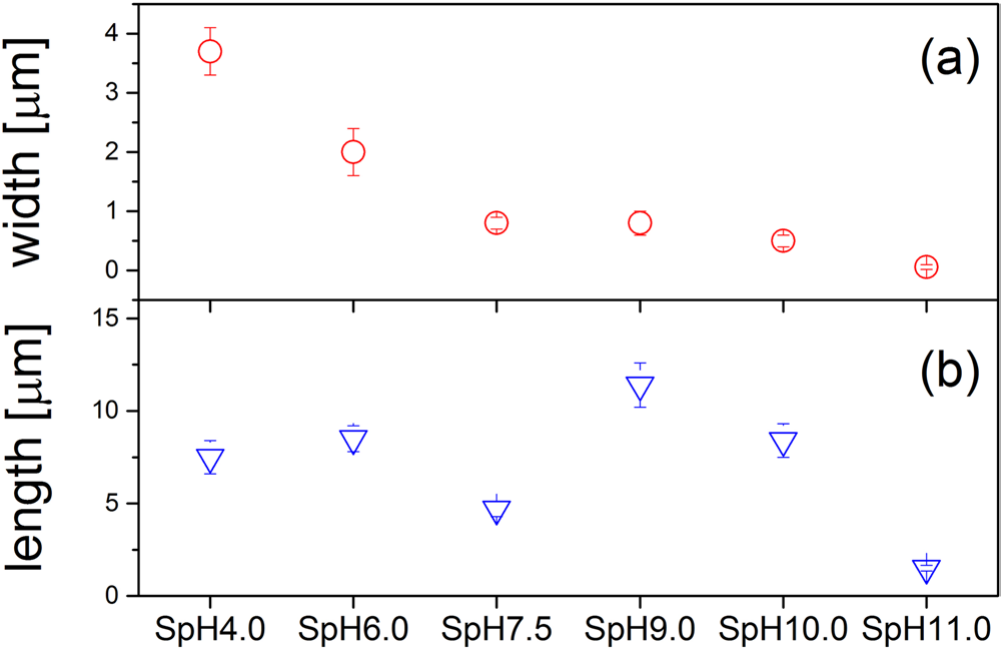
(**a**) Crystals width and (**b**) length determined from SEM images for particles obtained through EDTA/MEA-assisted hydrothermal synthesis at different pHs.

As a result of the hydrothermal treatment, we obtained powders containing micro- and nanometric crystals. Morphology and structure of the inorganic particles, which precipitated under high temperature and high pressure condition, were investigated as a function of MEA concentration. Monoethanolamine, which belongs to the amino alcohol group, stabilized the solution and allowed us to obtain homogeneous media in a wide range of pH from 4.0 to 11.0. When the calcium-phosphate solution was acidic, pH = 4.0, the obtained crystals had plate-like structure with smooth surfaces, as shown in Fig. 1a,b. The average width and length of the particle sheets, determined from SEM image, was approx. 4 μm and 7 μm, respectively. The increase of the concentration of MEA in the calcium phosphate solution with all other synthesis parameters kept at the same level resulted in the growth of different forms of crystals. Figure 2a–f shows SEM images of particles obtained in the pH range from 6.0 to 11.0. These micrographs show that particles formed as large irregular flakes with an average width of approx. 2 μm and length of approx. 9 μm when the synthesis was carried out at pH = 6.0 (Fig. 2a). When the synthesis was performed in pH = 7.5 these flakes structures remain but have slightly reduced dimensions (width of approx. 1 μm and length of approx. 5 μm) (Fig. 2b). When the pH was increased to pH = 9.0 and pH = 10.0 the bundles of rods appeared (Fig. 2c,d) with a similar morphology of crystals and with a comparable width of approx. 1 μm and length of approx. 10 μm. Finally, when we changed the conditions to pH = 11.0, the shape of the crystals substantially evolved resulting in generation of nanofibers (Fig. 2e,f). Within these nanostructures the nanofibers are characterized by a width approx. 100 nm and length approx. 1.5 μm, and their aspect ratio exceeds 10. Even though samples were repeatedly rinsed the fibre bundles were not destroyed, which can be seen from the SEM image (Fig. 2e,f). The evolution of crystal length and width with respect to MEA concentration is shown in the Fig. 3.

**Figure 4:**
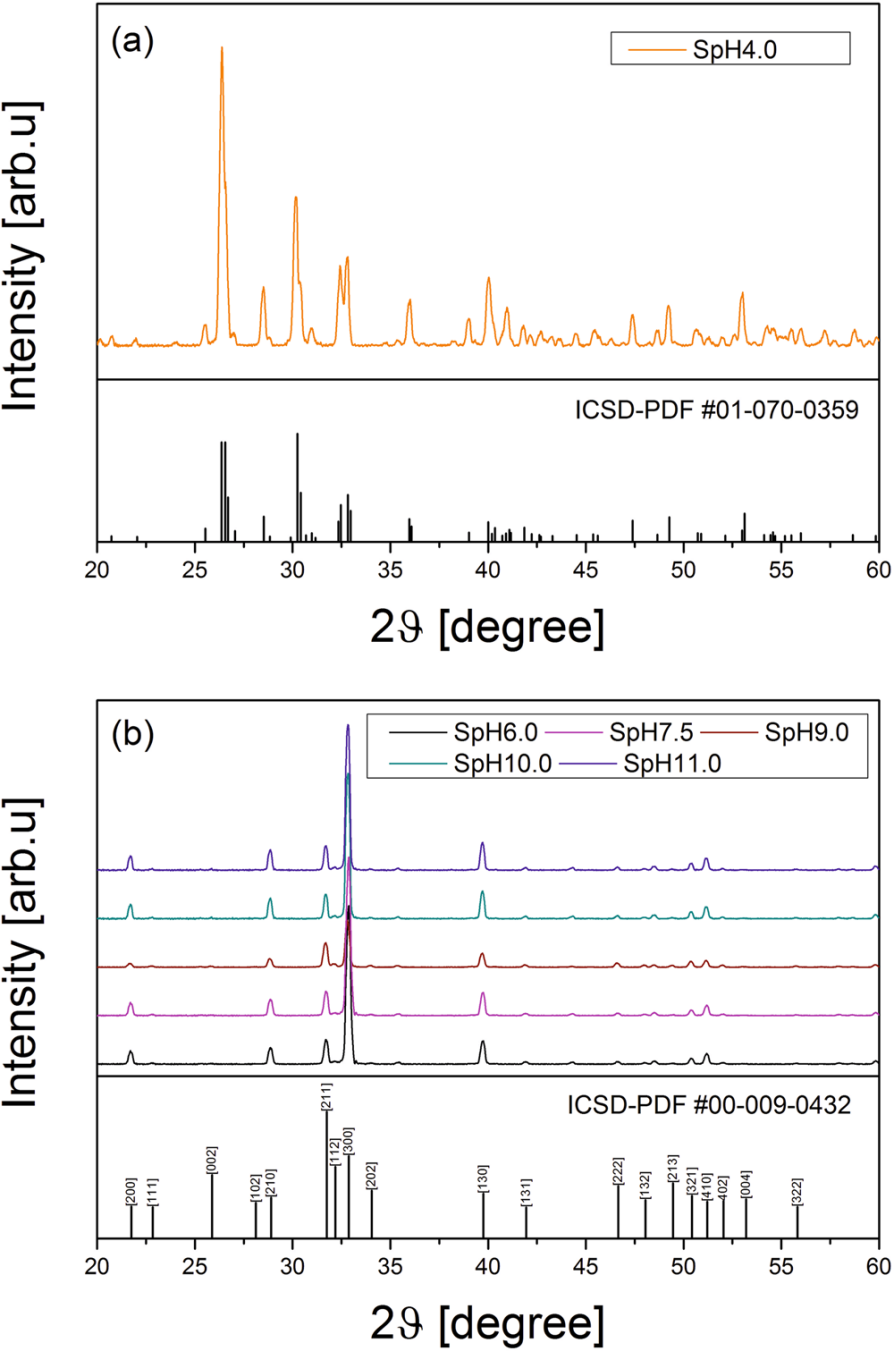
XRD patterns of precipitates obtained from calcium phosphate solutions with (**a**) pH = 4.0 and (**b**) pH ranging from pH = 6.0 to pH = 11.0.

XRD analysis of powders produced by the hydrothermal route is shown in Fig. 4. The diffraction pattern of the SpH4.0 specimen (Fig. 4a) can be assigned to a pure crystalline DCPA phase [space group *P1*], which is in a good agreement with the reference data (ICSD-PDF #01-070-0359). No peaks for any other phases were detected for this sample. The powder x-ray diffraction patterns (XRD) of other synthesized products are shown in Fig. 4b. Here, all diffraction peaks can be indexed and assigned to a pure HAp phase [space group: *P6*_3_*/m*] with calculated lattice constants a = 9.42(2) Å and c = 6.88(2) Å. The results of this analysis are consistent with the reference data (ICSD-PDF #00-009-0432). The XRD patterns for HAp particles displayed a stronger preference for (300) reflection (2θ = 32.9°) when compared to the reference data. This observation may be explained by the crystallographic texture^28,29^ or by preferred orientation of crystallites seen in Fig. 2. Anisotropy in hydroxyapatite crystal growth was observed by others and may results from hydrothermal process condition^30,31^.

The EDS elemental analysis was carried out to measure the calcium to phosphorus (Ca/P) molar ratio. The results including standard uncertainties were collected in Table 1. The results indicate that the Ca/P molar ratio for the SpH4.0 sample was approx. 1.07, which is close to the theoretical value of stoichiometric DCPA. For HAp powders synthesized at pH ≥ 7.5 (that is for the samples SpHx, where x = 7.5, 9.0, 10.0, 11.0), the EDS analysis revealed that the changes in the Ca/P molar ratio were within the margin of experimental error. The determined Ca/P value was approx. 1.67 and agrees well with the theoretical data on the stoichiometry of the HAp phase. For the sample SpH6.0 we observed a minor deviation from the theoretical value. A lower value of Ca/P molar ratio may suggest that the synthesized crystals were more defected, or that the hydroxyapatite was calcium deficient.

**Table 1:** Chemical composition of the calcium phosphate compounds measured by EDS.

sample name	SpH4.0	SpH6.0	SpH7.5	SpH9.0	SpH10.0	SpH11.0
MEA concentration [mol dm^−3^]	0.08	0.17	0.20	0.25	0.41	1.65
Ca/P molar ratio	1.07 (0.08)	1.55 (0.11)	1.65 (0.10)	1.68 (0.12)	1.67 (0.08)	1.70 (0.10)

**Figure 5:**
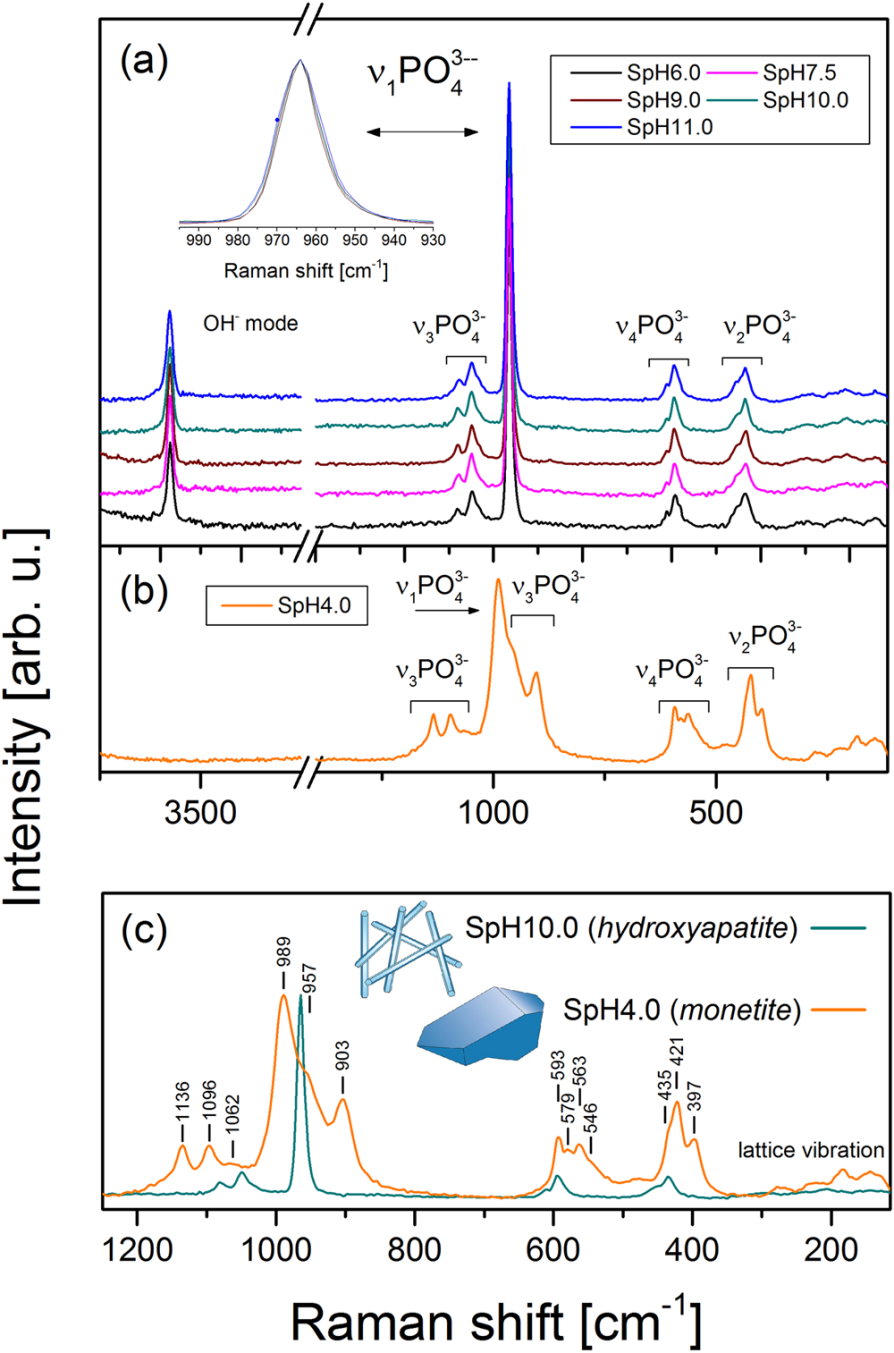
Raman spectra for hydrothermally synthesized powders. (**a**) Spectra of the particles synthesized at pH ranging from 6.0 to 11.0. (**b**) Spectra of the particles synthesized at pH = 4.0. (**c**) The comparison of the spectra obtained for DCPA and HAp.

To confirm the structural identification by XRD, we additionally performed a Raman spectroscopy study. The collected results are presented in Fig. 5. Figure 5a shows Raman spectra of powders precipitated at pH ≥ 6 (SpHx, x = 6.0, 7.5, 9.0, 10.0, 11.0), for which according to the XRD identification hydroxyapatite was obtained. Figure 5b shows the Raman spectrum obtained for the SpH4.0 sample. Figure 5c compares the Raman spectra obtained for DCPA and HAp (sample SpH4.0 versus SpH11.0) to highlight the structural differences between these two materials.

In general, for DCPA and HAp, the Raman vibrational spectra are dominated by the internal modes of PO_4_^3−^ tetrahedron. The free PO_4_^3−^ tetrahedron has *Td* symmetry, and its vibrational normal modes give rise to four different frequencies: (v1) mode arising from symmetric stretching of the P–O bonds of the tetrahedron (most intense peak), (v3) triply degenerate modes corresponding to the asymmetric P–O stretching involving also P motion, and finally doubly and triply degenerate mode corresponding to O–P–O bending deformations of the tetrahedron, ν2 and ν4, respectively^32^. The frequencies of the internal modes of an isolated PO_4_^3−^ tetrahedron are commonly quoted in the literature: these bands lie in the 400–1200 cm^−1^ spectral range^33^. The less intense bands positioned at 150–300 cm^−1^ are due to the external lattice modes. The positions and assignments of bands for the free PO_4_^3−^ tetrahedron were collected in Table 2.

**Table 2:** Observed Raman frequencies at room temperature for DCPA (monetite) and HAp (hydroxyapatite) together with band assignment.

Assignment	Band position [cm^−1^]		*Band position for free PO_4_^3−^ [cm^−1^]
	DCPA	HAp	
lattice modes	150–300	150–300	—
OPO bend, v2	397	433	420
	421	454	
	435		
OPO bend v4	546	589	573
	563	596	
	579	613	
	593		
P-O str., v3	903		1004
	957		
P-O str., v1	989	964	936
P-O str., v3	1062	1033	1004
	1096	1049	
	1136	1079	
v(OH)	—	3575	—

The internal modes of the free PO_4_^3−^ group are given for comparison*^34^.

In Fig. 5a, we see that for all samples synthesised at pH ≥ 6.0 Raman spectra contain the same features that are characteristic for HAp phase^29^. The spectra are dominated by the internal mods of the PO_4_^3−^ tetrahedron (as was previously described); however, the observed frequencies are blue shifted (by about 20 cm^−1^) in reference to the normal modes of an isolated PO_4_^3−^ group (see Table 2). This indicates that there is a strong crystal field in the structure leading to distortion of the spectral lines; this effect was also observed by other authors^29^.

The spectrum collected for SpH4.0 particles (Fig. 5b) can be assigned to the DCPA phase according to the available literature data^34^. Figure 5c shows comparison of spectra obtained for SpH4.0 and SpH11.0, where we can see that spectra assigned to the DCPA and HAp phase are visibly distinguishable. We observed the distinct shifts and splitting of the PO_4_^3−^ vibrational frequencies in DCPA spectra in relation to the normal modes of the isolated phosphate ion. Similarly to HAp this results from the presence of the local crystalline site field created by the surrounding ions, which distorts the PO_4_^3−^ tetrahedron in DCPA structure, reduces the symmetry, and gives rise to the splitting of the degenerate modes of the tetrahedron. Greater complexity of the DCPA spectrum suggests the presence of an additional effect affecting the vibrational properties of the DCPA crystals. According to Kravitz *et al*. and Casciani *et al*.^34,35^, this may result from the existence of cooperative vibrations in which the neighbouring PO_4_^3−^ ions are coupled together and give rise to the so-called correlation field splitting. To summarize, our Raman spectroscopy results confirm the XRD study and can be used to distinguish different phases of calcium phosphates.

## Discussion

Micrometric monetite crystals and micro- and nanometric HAp crystals were formed in a solution containing EDTA and MEA under hydrothermal conditions. For low concentrations of MEA (0.08 mol dm^−3^), we obtained DCPA phase. With an increase of the MEA content (above 0.08 mol dm^−3^) hydroxyapatite particles were produced with the Ca/P molar ratio relatively close to the stoichiometric value of HAp. The crystal morphology evolved from the irregular plates, through aggregates of hexagonal rods, to the nanofiber shape. With an increase of pH we observed a decrease in the aspect ratio of HAp crystals. In general, for HAp crystals hexagonal close packed symmetry imposes that the most energetically advantageous crystal growth occurs along [001]. This is because the {001} planes have higher surface free energy than {100} planes^36^. This leads to the formation of hexagonal rods. However, when the growth kinetics is disturbed (e.g. by organic additives), a diffusion-limited growth occurs, with crystal growth rate far beyond the equilibrium state. This ultimately leads to the reduction of certain crystal faces or even to the crystal disappearance^37,38^.

Although the hydrothermal procedure is relatively simple, the actual mechanism of the formation of different phases and shapes of calcium phosphates remains an open question. Nevertheless, based on the presented experimental results, we propose two factors determining the final phase and morphology of calcium phosphate crystals, which are the organic additives: (i) calcium chelating reagent EDTA and (ii) amino-alcohol MEA as the stabilizer and pH adjusting compound. The temperature and synthesis time, which also play an important role in nucleation and crystal growth under hydrothermal conditions, remained fixed in all cases so their impact is not discussed.

EDTA is a strong chelating agent that has a distinct effect on the morphology of different crystals formed by wet chemical methods^39^. It controls the release of calcium ions into the solution and hence the degree of supersaturation. At room temperature, the hexadentate ligand combines with calcium ions. The hydrothermal conditions, i.e. high temperature and high pressure, shift the chemical equilibrium of the Ca-EDTA complex towards its dissociation. The chelation of the complex weakens, and Ca^2+^ ions are released into the solution. When the ionic activity product in the solution exceeds the thermodynamic solubility product, nucleation and a subsequent crystal growth occurs.

**Figure 6:**
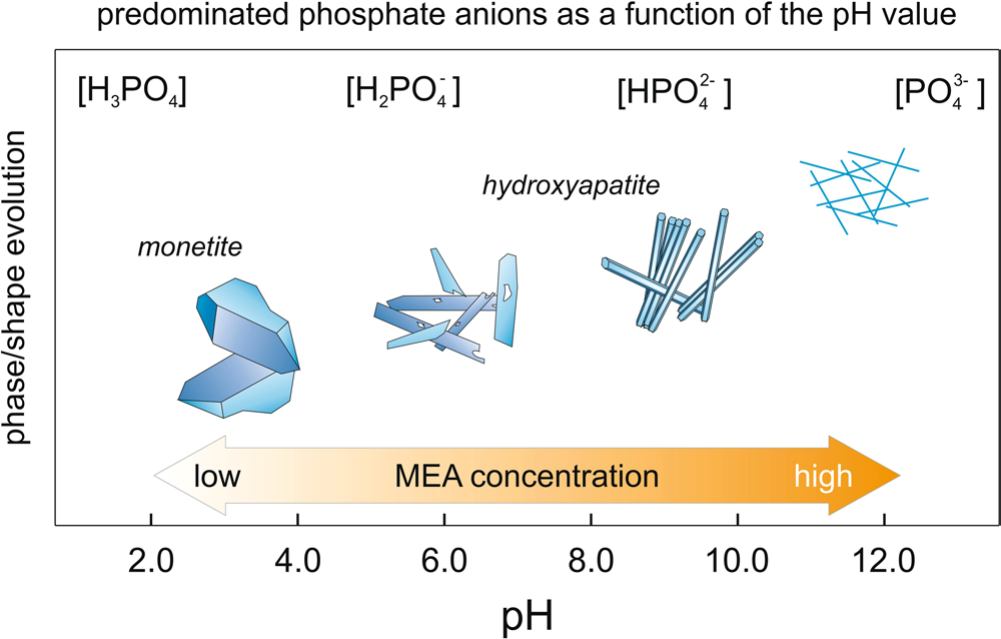
Schematic illustration of morphological and structural evolution of calcium phosphate particles during the hydrothermal process.

On the other hand, we also have MEA present in the solution, thanks to which we manage to create a stable solution in a wide pH range, i.e. from 4.0–11.0. MEA is a water-soluble primary amine and coordination agent, used for the synthesis of various nanomaterials^27,40^ and to examine its effect on the morphology of synthesized particles^41^. However, the effect of MEA on the morphology of calcium phosphates synthesized through hydrothermal method has not yet been studied. Figure 6 shows shape and phase evolution of the synthesized particles together with the increasing concentration of MEA (from 0.08 mol dm^−3^–1.65 mol dm^−3^). It is known that local supersaturation causes the precipitation of calcium phosphates with low solubility product and high thermodynamic stability^37^. The type of the synthesized material strongly depends on the pH. According to the solubility phase diagram for calcium phosphates, at pH lower than 4.2, the least soluble (most stable) salt is the DCPA, while for pH values greater than 4.2, hydroxyapatite is the most thermodynamically stable phase^37^. The chemical composition and Ca/P molar ratio of the synthesized products are also integrally linked to the amount of orthophosphate species in the solution, which in turn are affected by the pH (as shown in Fig. 6). The dihydrogen phosphate ions are stable at 3 < pH < 6. If the local pH increases to between 8.0 and 11.0, HPO_4_^2−^ ions predominate, whereas beyond pH = 12.0 the PO_4_^3−^ phosphate ions occur. Our results are in line with these considerations. For low concentration of MEA, the DCPA is formed accordingly to the following chemical reaction:

$${{\rm{Ca}}}^{2+}+{{\rm{H}}}_{2}{{\rm{PO}}}^{4-}\to {{\rm{CaHPO}}}_{4}+{{\rm{H}}}^{+},$$

while for MAE concentrations above 0.08 mol dm^−3^ HAp is formed:

$$10{{\rm{Ca}}}^{2+}+6{{{\rm{PO}}}_{4}}^{3-}+2{{\rm{OH}}}^{-}\to {{\rm{Ca}}}_{10}{({{\rm{PO}}}_{4})}_{6}{({\rm{OH}})}_{2}.$$

However, at pH = 11.0, when MEA concentration is as high as 1.65 mol dm^−3^, we observe a visible reduction in HAp crystals size, while maintaining the purity of structure. The viscosity of MEA is an order of magnitude greater than water (20 mPa s vs 0.89 mPa s) and the change in pH from 10 to 11 required almost four times more MEA than the change from 9 to 10. As a result of the increased MEA concentration the viscosity and density of the calcium phosphate solution increases. On the other hand, we know that the diffusion of atoms or molecules in liquid medium depends, among other things, on the dynamic viscosity^42^. Therefore, we postulate that when the solution pH is greater than 10.0, a limited diffusion of crystal building compounds appeared leading to the crystal growth inhibition. Another effect that can be triggered by the organic additives, such as MEA, is the preferential adsorption of additive molecules and ions at different crystal faces, which could induce the crystal growth rate in different crystallographic directions^43^.

## Conclusion

In the present study, we propose a new facile strategy for synthesizing different phases of calcium phosphates. The presented results show that hydrothermal synthesis in the presence of EDTA and MEA (instead of urea or ammonia) leads to the formation of pure phase monetite and HAp structures. The use of MEA allowed to obtain a stable and homogeneous solution in a wide pH range. We showed that changes in the MEA concentration within solution affect the hydroxyapatite morphology and lead to the formation of HAp microcrystals and nanofibers.

## Methods

### Synthesis scheme

All reagents were analytical grade. In a typical synthesis procedure, calcium nitrate Ca(NO_3_)_2_ (0.2 mol dm^−3^) and diammonium phosphate (NH_4_)_2_HPO_4_ (0.12 mol dm^−3^) were individually dissolved in ultrapure water (18.2 MΩ cm^−1^) from a Polwater system. EDTA was added to the solution containing calcium source as a chelating agent to obtain a fixed molar ratio of EDTA:Ca^2+^ (1:1). Afterwards, MEA was dissolved in the Ca-containing solution. The molar concentration of MEA was set to a specific value ranging from 0.08 to 1.65 mol dm^−3^. When all reactants were completely dissolved both solutions containing calcium and phosphorous precursors were combined together and subsequently stirred at room temperature for 30 min. The final homogenous solution was transferred into 200 ml Teflon vessel which was placed in a hydrothermal reactor (Carl Roth 2098.1). The autoclave was sealed and maintained at 200 °C for 7 h. The system was then cooled to the ambient temperature. The resulting suspension was centrifuged (5000 rpm, 5 min) and washed with deionized water to remove the residual MEA until the pH of the filtrate was neutral. The obtained precipitates were labelled as SpHx, where x denotes the initial pH value of solution before the hydrothermal reaction (x = 4.0, 6.0, 7.5, 9.0, 10.0, 11.0). All results have been checked for repeatability.

### Characterization

The morphology of synthesized particles was examined using scanning electron microscope (SEM, Tescan Vega 3). We analysed SEM images to determine the size of the obtained particles. Briefly, we chose the ROI of approximately 40 × 40 μm for each SEM image. Next, the typical crystals (about 20 pieces for each sample) with well-defined boundaries were identified within ROI and imageJ software was used to automatically determine the average width and length of the crystals and to calculate the standard deviations. The elemental analysis was performed using energy dispersive x-ray spectrometer (QUANTAX EDS, Bruker) equipped with an XFlash 610 M detector with the resolution of <129 eV for the Mn Kα line. The crystal structure was determined with the x-ray diffractometer (XRD, PANalytical X’Pert Pro) using standard θ–2θ geometry. The detection was performed using the Cu K_α_ (λ = 1.54 Å) radiation at operating current and voltage of 30 mA and 40 kV, respectively. The angular resolution of the instrument was calibrated using LaB_6_ line profile standard (SRM660a–NIST certificate). The chemical composition of synthesized particles was determined with the Raman spectrometer (Almega XR of Thermo Electron Corp.). The chosen excitation light wavelength was 532 nm. Data was recorded in the spectral range from approx. 100 cm^−1^ up to 4000 cm^−1^ and with the spectral resolution of 2 cm^−1^.

## Data Availability

Data generated and analysed in this study are available from the corresponding author upon request.
